# A social problem analysis of the 1993 Brady Act and the 2022 Bipartisan Safer Communities Act

**DOI:** 10.3389/fpubh.2024.1338722

**Published:** 2024-03-27

**Authors:** Devon Ziminski

**Affiliations:** ^1^School of Social Work, Rutgers University, New Brunswick, NJ, United States; ^2^New Jersey Gun Violence Research Center, School of Public Health, Rutgers University, Piscataway, NJ, United States

**Keywords:** firearm injury prevention, gun violence, content analysis, social problem awareness, issue definition, federal policy making, social problem analysis

## Abstract

In June 2022, the U.S. federal government passed its first major firearm policy since the Brady Handgun Violence Prevention Act of 1993, the Bipartisan Safer Communities Act (BSCA). Summative content analysis was used to explore how the social problem of firearm violence was outlined in both policies, with the goal of extracting the social issue’s definition from the policies’ approaches to solving it. Both policies do not outline the various types of firearm violence, nor the disproportionate effect of firearm violence on certain populations. This work informs the role of federal policy in defining and monitoring firearm violence as a public health issue, identifying both individual and structural risk and protective factors from an asset-based lens, and allocating preventative efforts in communities that are most affected.

## Introduction

Approximately 100,000 Americans are shot and injured, and over 1,800 children are killed each year by firearms (1). Firearm violence results in the loss of life and induces immense trauma in the lives of individuals and in communities where tragedies occur. As firearm violence continues to affect communities across the U.S., there remains a need to appropriately and collectively define the social problem to draw aligned conclusions and support equitable appropriations in subsequent policies. Scholars (2–4) have pointed to the importance of social issue framing within the of the advocacy and policy processes yet note that focus on social problem definition and positionality is limited in existing public health literature. Issue definition, the way social problems and their policy issues are understood, is critical for public discourse and legislative processes (5–7). The current view of the problem of firearm violence as it’s outlined in policies (federal and local) does not always align with the daily realities of firearm violence’s toll. This brief presents a social problem analysis of the two most comprehensive U.S. federal firearm policies in the last 30 years (The Brady Handgun Violence Prevention Act (8) – Brady Act and The Bipartisan Safer Communities Act – BSCA) and the ways that the policies broadly and specifically focus on populations affected by firearm violence.

### Policy background

The goal of this work is to outline the social problem of firearm violence, consider the ideological frameworks that undergird the Brady Act and BSCA, and explore how the policies’ text may underemphasize the social realities of U.S. firearm violence. Key takeaways focus on extracting the social issue’s definition from the policies’ approaches to solving it; generating a shared understanding of the problem of firearm violence, including its varying types and who is affected; and identifying ways to support equity in future federal firearm policy interventions and their implementations.

The social problem of firearm violence represents a complex and sometimes divisive area of inquiry due to deep seated political beliefs, cultural norms, and existing policies. Despite this, the social problem of firearm violence permeates American life, warranting a comprehensive outline and understanding of the scope of the social issue [(9), 6]. And this issue is deepening, with firearm violence increasing over the recent decades [(10), 1–3; (11), 4]. Despite the continuing violence, there at times exists a disparity in how firearm violence is conceptualized publicly and outlined in federal policy.

In any conversation on firearm violence, it is critical to denote the various types of firearm violence as they are often conflated, yet each type has distinct causes, consequences, populations affected, and solutions. Firearm violence is violence that occurs with the use of a firearm, which includes homicide (including community violence and mass shootings), other interpersonal violent crimes (e.g., gun assaults, non-fatal shootings), police violence, intimate partner violence, suicide and attempted suicide, and unintentional death and injury (12). In 2021, 54 percent of all firearm-related deaths in the U.S. were suicides (26,328), 43 percent were homicides (20,958) (13, 14), and the remaining firearm deaths were unintentional (549), involved law enforcement (537), or had undetermined circumstances (458). Suicides by firearm most often occur among often older white men, while firearm homicides are disproportionately concentrated among young Brown and Black men [(1, 15), 3–6]. Black people are 10 times more likely, and Hispanic/Latino residents are more than twice as likely, to be killed by firearm than white residents (1, 13, 14). Firearm violence is the leading cause of death for Black males ages 15–34 (1, 13, 14).

There are multiple determinants of firearm violence, predominantly rooted in structural inequities and intentional disinvestment in certain places and among groups of people, which cultivates conditions for intrapersonal, interpersonal, and/or community (among non-intimately involved individuals) Firearm violence to occur. Many of the neighborhoods affected by firearm violence are also affected by systemic inequities (e.g., income inequality, poverty, and underfunded public housing and public/social services), with these inequities stemming directly from racism [(16–18), 833–841]. As such, many deep social structural disadvantages combined with easy access to guns (both legal and illegal) can contribute to varying conditions of firearm violence [(19), 85–90; (20, 21)]. The most sweeping federal firearm policy prior to the Brady Act was the 1968 Gun Control Act (GCA) that followed the assassinations of President John F. Kennedy, Attorney General and U.S. Senator Robert F. Kennedy, and Reverend Dr. Martin Luther King, Jr. The GCA repealed and replaced the Federal Firearms Act of 1938 (22). Following the 1981 assassination attempt of President Ronald Reagan where White House Press Secretary Jim Brady was also gravely injured, Jim and Sarah Brady led a multi-year long effort that led to the 1993 passage of the Brady Bill. The Brady Act amended the GCA and required state and local law-enforcement officials to perform background checks during the five-day waiting period before a federally licensed firearms dealer (FFL) could sell a handgun to a potential purchaser (23). In 1998, the electronic National Instant Criminal Background Check System (NICS) debuted to aid in the processing of background checks (23, 24).

The most substantial piece of federal firearm legislation since the Brady Act passed Congress in June 2022 – the Bipartisan Safer Communities Act (BSCA). Spurred into action by the horrific mass shootings in Buffalo, NY and Uvalde, Texas in May and June 2022, the Act is both a reaction to public outcry and an example of quick bipartisan effort and collective action. This legislation expanded resources for crisis intervention programs including red flag laws (known as Extreme Risk Protection Orders - ERPOs), which allow law enforcement or courts to temporarily take firearms from someone believed to be a danger to themselves or others (25, 26). The legislation also requires more people who sell guns as primary means of income to register as FFL dealers, encourages states to include juvenile records in the NICS, and denies access to firearms (for 5 years) for people who are convicted of [committing] violence while in dating relationships.

### Policy analysis

In evaluating the alignment of the social problem of firearm violence with the two most recent comprehensive federal firearms policies, the Brady Act and BSCA, two main aims are: identify the problematization of firearm violence, and explore how the purported problem framing and definitions may align or diverge from the realities of U.S. firearm violence [(27), 63–70], both within and between the two policies.

A summative content analysis (through NVivo14) was used to explore how the social problem of firearm violence was outlined in both the Brady Act and BSCA S.1536, (8, 28). The summative approach directly assesses text to understand the contextual use of its words (7, 29). Codes were developed based on established terms associated with social issue definitions from the policy analysis literature (7, 30–32) and words were coded by the author. The four codes to assess the social problem definition included causes, context, and contributing factors; definitions; populations affected; and magnitude. Because neither text had an explicit section outlining the social problem or issue definition, the developed codes highlighted elements of the policy that refer to any components of a problem definition. Words and phrases categorized related to the issue framing were examined for the four areas of: any phrases that discussed the context of where or how firearm violence happens; any explicit phrasing of any definition of a term in the text; any text referencing a specific group of people or population(s); and any text referencing; and any text noting the magnitude or size of the issue of firearm violence (in people, cost, or other numbers). References in texts were tabulated within each text and between the two texts, and reference percentages were compared by the combination of words and phrases that accumulated for each code. Validity was increased by multiple readings and inclusion of multiple codes for the capturing the inclusion or outline of a problem definition in each text.

The Brady Bill had three coded references across the entire document, a mere 5.6% of the policy’s content, and the BSCA had 31 coded references across the document, one-fifth of the available text (19.3%). Of note here are the low percentages of coded references in each text, very limitedly capturing two of four codes both in and between the two texts. The four problem definition classifications overall did not work well for the two texts because the problem definition and related concepts around the social issue were not included in the texts, hence the multiple 0%s of the 267 and 2,543 reference coded words seen in Table 1. We might expect some supporting background around firearm violence definitions in policies, yet this information is not explicitly included in the Brady Bill or BSCA.

**Table 1:** Within policies explicit coded words.

	Brady Act (4,738 total words)	BSCA (13,209 total words)
Total reference coded words (267 and 2,543 words coded)	5.6% (267 coded words)	19.3% (2,543 coded words)
1: Causes, Context, & Contributing Factors	0% (0 of 267 coded words)	0% (0 of 2,543 coded words)
2: Definitions	55.81% (149 of 267 coded words)	72.04% (1,832 of 2,543 coded words)
3: Population(s) Affected	44.19% (118 of 267 coded words)	27.96% (711 of 2,543 coded words)
4: Magnitude	0% (of 267 coded words)	0% (0 of 2,543 coded words)

In looking at the codes associated with defining or understanding the social problem – we see that both policies have 0% references to causes, context, and contributory factors, and magnitude of the problem. In the Brady Bill, the 55.81% (two references coded) for definition are with regards to definitions of “handgun” and “licensee.” The 44.19% (one reference coded) for population refers the population of prohibited persons (“a fugitive from justice; an unlawful user of or addicted to any controlled substance; has not been adjudicated as a mental defective or been committed to a mental institution; is not an alien who is illegally or unlawfully in the US; has not been discharged from the Armed Forces under dishonorable conditions; and is not a person who has been a citizen of the US, and renounced such citizenship,” p3) which helps identify the populations deemed incapable of firearm ownership or use.

While 72.04% (20 phrases coded) in BSCA falls under the definition code, these references are not directly related to the problem definition of what firearm violence is but define concepts such as “engaged in the business,” “drug trafficking crime (used to explain straw purchasing), “dating relationship,” and also outline the parameters of the established Federal Clearinghouse on School Safety Evidence-based Practices. The elucidation of “trafficking crimes” and “dating relationship” (as it pertains to people who may engage in abusive behavior) further explains the types of prohibited persons that are barred from firearm ownership or use, and contributes to the policy’s outlining of these individuals being in some way potentially associated with the occurrence of firearm violence.

Similarly, the population code (27.96%, 11 phrases coded) in BSCA refers to populations that are mentioned in the bill that are either receiving funds/support or identified in the bill for another reason as part of the policy intervention. For example, Sec.11002, Medicaid and Telehealth, refers to improving medical services “including addressing the needs of individuals with disabilities, medically underserved urban and rural communities, racial and ethnic minorities such as American Indians and Alaska Natives, individuals with limited English proficiency, and individuals of different age groups including children, young adults, and seniors” (p5). While this phrasing points to where resources from the policy might be allocated, no individuals or groups were noted with relation to the social problem of firearm violence or who it affects. The following words were not included (or defined) in either texts: “firearm injury/injuries,” “mass shooting,” “gun violence,” and firearm violence.”

Ideally a problem analysis would reveal framing or definitions around historical context and determinants of a social problem, which inherently are often laden with values and ideologies in how a problem is both conceptualized and thus defined. For instance, the background section of this article outlined firearm violence through structural and systemic factors, and identified people who are disproportionately affected by the issue. The term and phrase definitions included in Brady and BSCA mainly focused on individuals (prohibited persons) who may be presumed to be associated with firearm violence but did not outline how or to whom this violence happens. Oftentimes, without a social problem’s explicit denotation in text, readers are left to deduce underlying theories and relational assumptions about what problems exist and for whom they exist, coded through implicit language and phrases (31, 32). As such, for this analysis, implicit language around the problem of firearm violence can be chronicled through textual examples that highlight values and relational assumptions around firearm violence, as no direct mentions to the social problem are included.

Implicit values around individual firearm rights are reinforced in both Brady and BSCA through specific provisions. For example, the Brady Bill text provides procedures for rectifying wrongful denial of a firearm background check (Sec. 108.); and in the BSCA, detailed procedures for due process in Extreme Risk Protective Orders cases (Sec. 12,003), 5-year time limits on domestic abuser firearm restrictions (Sec. 12,005), and sunsetting of inclusions of juvenile records in the NICS after 10 years (n, SEC. 12,001) are included. Both texts include specific references to restrictions on the establishment of a federal system of registration of firearms. Furthermore, underlying theories and assumptions related to firearm violence are largely veiled as criminality of prohibited persons in the Brady Act and shift to additional individual criminal acts in BSCA (e.g., straw purchasers, those convicted of domestic violence), with mental illness also implicitly being noted as a driver of violence. For example, this relational assumption in BSCA is that increasing mental health resources and law enforcement in schools may reduce firearm violence (Subtitle C – Luke and Alex School Safety Act of 2022). While there are complex interplays to consider when discussing criminal intent, deterrence methods, and mental illness, the connection drawn between these concepts as both potential an all-encompassing cause and panacea to firearm violence is insufficient and can easily be misconstrued [(33), 31; (34), 275–282].

A key limitation to this policy analysis is the differing lengths of the two policy texts, hence the higher coded percents in BSCA compared to Brady Bill in Table 2. Another limitation is the single coder, introducing a lack of inter-coder reliability and no explicit review of codes by content experts (29). Additionally, traditional policy analyses review a larger number of documents (e.g., congressional hearings) (7, 35). Future analyses should include multiple coders, more specific definitions of codes, and expand the number of federal and/or state firearm policies included in analysis.

**Table 2:** Between policies explicit coded words.

	Brady Act (4,738 total words)	BSCA (13,209 total words)
Total reference coded words (2,810 words coded)	9.5% (267 words coded)	90.5% (2,543 words coded)
1: Causes, Context, & Contributing Factors	0% (0 of 2,810 coded words)	0% (0 of 2,810 coded words)
2: Definitions	7.52% (211 of 2,810 coded words)	92.48% (2,599 of 2,810 coded words)
3: Population(s) Affected	14.23% (400 of 2,810 coded words)	85.77% (2,410 of 2,810 coded words)
4: Magnitude	0% (0 of 2,810 coded words)	0% (0 of 2,810 coded words)

### Actionable takeaways

The stated goals of the Brady Act were: to “provide for a waiting period before the purchase of a handgun, and for the establishment of a national instant criminal background check system to be contacted by firearm dealers before the transfer of any firearm,” and for BCSA: to “make our communities safer.” The goal of any bill is to outline policy addressing a social problem, and not necessarily to outline the problem itself; however, social problem definition remains essential to lay context for a bill’s goals and can both support issue framing and provide implementation direction, particularly among social issues that are contentious like firearm violence (2). Both policies explored conflate all firearm violence by neither addressing the types of firearm violence nor who is most affected by each or any type. Issue definition absence can muddle policy options or lead to claims of policy ineffectiveness when policies are not explicit about who or what social issue they aim to address. Also, it can be challenging to re-frame and disseminate definitions once existing frames are in play, further emphasizing the importance of identifying definitions and circulate it widely to increase awareness (5, 6, 36).

Firearm violence is mainly driven by suicides and then homicides. Daily incidents of firearm suicide, community firearm violence, and intimate partner firearm violence affect young Black and Brown men, older men, rural populations, veterans, LGBTQIA+ people (especially transgender people of color), and people affiliated with certain religions, disproportionately - and these disparities in firearm violence can often be overlooked or melded together [(10, 15, 17, 37–41), 154–167]. To contribute to both narrative and tangible change, the distinct effects of firearm violence can be better elucidated in public discourse and records - especially in policy. The research provided, resources needed, and policies proposed to address suicide among older white men will be different than intimate partner firearm situations within heterosexual romantic relationships, which will be different than community firearm violence involving young Black men.

Both policies allude to individual criminal acts or mental state as key drivers of firearm violence; excluding, among multiple other drivers, the structural elements and historical inequities that contribute to firearm violence [(42), 224–241; (43), 165; (44), 253–266]. The absence of socioecological context, how it connects social determinants of health and firearm violence, and mention of who is most disproportionately affected by each strain of this social issue distances both policies from the issue’s causes and contexts, potentially from its solutions rooted within the realities of people and communities who are affected (31, 32, 45, 46).

The Brady Act’s and BSCA’s vagueness around firearm violence could be perceived as a political maneuver for claims of future policy ineffectiveness or ambiguous accountability associated with their provisions, as examples of imperfect yet essential compromises in political process, or a combination of both. Regardless, how policy goals are operationalized through allocation, delivery, and finance will be critical for BSCA implementation in 2024 and 2025, a task assigned to the recently created White House Office of Gun Violence Prevention.

Federal policies can continue to move toward defining and monitoring the problem of firearm violence at the policy level in ways that align with the problem’s magnitude (35). The complexity of this issue warrants a widely purported problem definition and dissemination to spur sustained action (12). A movement toward asset-building and resource investment in communities as a public health approach, public safety model, and firearms violence solution remains needed [(47), 201–230; (48, 49); 2,169–2,178]. To support this outcome, the research community can continue to engage in research translation toward more pointed policy and practice approaches.

## Conclusion

Issue definitions should aim to outline the realities of social problems. The Brady Act and BSCA conflate various types of firearm violence, illustrating a focus on individual level factors without inclusion of structural elements that contribute to the critical socioecological context of self-inflicted, interpersonal, and community firearm violence.

## Author contributions

DZ: Conceptualization, Formal analysis, Methodology, Writing – original draft, Writing – review & editing.
